# Genetic analysis and prenatal diagnosis of short-rib thoracic dysplasia 3 with or without polydactyly caused by compound heterozygous variants of *DYNC2H1* gene in four Chinese families

**DOI:** 10.3389/fgene.2023.1075187

**Published:** 2023-03-17

**Authors:** Yuying Fang, Shuo Li, Dongyi Yu

**Affiliations:** ^1^ Center for Medical Genetics and Prenatal Diagnosis, Key Laboratory of Birth Defect Prevention and Genetic, Medicine of Shandong Health Commission, Key Laboratory of Birth Regulation and Control Technology of National Health Commission of China, Shandong Provincial Maternal and Child Health care Hospital affiliated to Qingdao University, Jinan, Shandong, China; ^2^ Genetic Testing Center, Qingdao Women and Children hospital, Qingdao, Shandong, China

**Keywords:** short-rib thoracic dysplasia 3 with or without polydactyly, *DYNC2H1*, whole-exome sequencing, compound heterozygous variants, prenatal diagnosis

## Abstract

**Background:** To describe the genetic variation of dynein cytoplasmic 2 heavy chain 1 (*DYNC2H1*) gene in four Chinese families affected with short-rib thoracic dysplasia 3 with or without polydactyly (SRTD3), and to provide evidence for accurate prenatal diagnosis and genetic counseling.

**Methods:** The detailed clinical prenatal sonographic features of four fetuses with SRTD3 were carried out. Trio-whole exome sequencing (WES) and proband-WES sequencing was applied to filtrated causative variants in four families. The causative variants of each family were validated in by Sanger sequencing. Bioinformation analysis was applied to predict the harmfulness of these mutations and perform the protein-protein interaction network and Gene Ontology (GO) analysis. A vitro minigene splicing assay was conducted to assess the influence of the splice site variant.

**Results:** Typical characterization of the four fetuses included short long bones, short ribs, narrow chest, hand and foot posture abnormalities, femur short in diameter and slightly bowing, cardiac abnormalities, and so on. Moreover, eight compound heterozygous variants of *DYNC2H1* (NM_001080463.2): c.3842A>C (p.Tyr1281Ser) and c.8833-1G>A, c.8617A>G (p.Met2873Val) and c.7053_7054del (p.Cys2351Ter), c.5984C>T (p.Ala1995Val) and c.10219C>T (p.Arg3407Ter), c.5256del (p.Ala1753GlnfsTer13) and c.9737C>T (p.Thr3246Ile), were identified. Among which, c.10219C>T (p.Arg3407Terp), c.5984C>T (p.Ala1995Val) and c.9737C>T (p.Thr3246Ile) were reported in ClinVar databases, and c.8617A>G (p.Met2873Val), c.10219C>T (p.Arg3407Ter), c.5984C>T (p.Ala1995Val) were found in HGMD databases. Four variants (c.3842A>C (p.Tyr1281Ser), c.8833-1G>A, c.7053_7054del (p.Cys2351Ter) and c.5256del (p.Ala1753GlnfsTer13) were first reported as novel mutations. According to the ACMG guidelines, c.8617A>G (p.Met2873Val), c.7053_7054del (p.Cys2351Ter), c.5984C>T (p.Ala1995Val), c.10219C>T (p.Arg3407Ter) and c.5256del (p.Ala1753GlnfsTer13) were rated as pathogenic or likely pathogenic variants, others variants were predicted to be variants of uncertain significance mutations. The minigene assay results indicated that c.8833-1G>A caused the skipping over exon 56, resulting in exon 56 loss.

**Conclusion:** In our study, we analyzed the genetic mutations in four fetuses with SRTD3 by whole exome sequencing and identified pathogenic variants causing SRTD3. Our results expand the mutation spectrum of *DYNC2H1* in SRTD3, which is helpful for the accurate prenatal diagnosis of SRTD3 fetuses and provide useful strategies for genetic counseling.

## Introduction

Skeletal dysplasia is a group of rare genetic disorders associated with abnormalities of cartilage and bone (Victoria et al., 2018). Genetic skeletal disorders are clinically and genetically heterogeneous with more than 350 genes accounting for the diversity of disease phenotypes (Bonafe et al., 2015). Despite great advances in imaging technology, fetal skeletal dysplasia is difficult to diagnose *in utero* because of its large number and overlapping phenotypic features. Prenatal diagnosis of skeletal dysplasia is particularly challenging due to highly variable but limited prenatal phenotypes.

Currently, short-rib polydactyly syndromes is classified as short-rib thoracic dysplasia with or without polydactyly types 1–17 (Loo et al., 2021). Short-rib thoracic dysplasia 3 with or without polydactyly (SRTD3, OMIM: 613091) covers a range of autosomal recessive or digenic recessive skeletal dysplasia characterized by shortened limbs, a narrow trunk, and associated visceral abnormalities with or without polydactyly (Chen et al., 2018; Cheng et al., 2022). Homozygous or compound heterozygous mutations of dynein cytoplasmic 2 heavy chain 1 (*DYNC2H1*) gene usually lead to SRTD3 (Mei et al., 2015; Chen et al., 2016; Xia et al., 2021; Zhao et al., 2022).

To date, more than 140 variants in the *DYNC2H1* gene have been found in SRTD3 (Chen et al., 2016). Mutations in *DYNC2H1* will cause the dysfunction of primary cilia, causing a heterogeneous spectrum of conditions such as skeletal dysplasia (Okamoto et al., 2015; Chen et al., 2016). Whole exome sequencing (WES) has been shown to be a powerful mean for prenatal diagnosis. Nevertheless, the different etiology of ciliopathies and extensive genetic variation lead to phenotypic variability. Thence, it is essential to fully understand the prenatal phenotypes of different ciliopathic syndromes before performing WES assays.

In our study, we analyzed gene mutations in four SRTD3 fetuses by WES and determined that the compound heterozygous mutation of *DYNC2H1* was the causative mutation of SRTD3. In this study, we found typical and atypical features of four fetuses with pathogenic variants in *DYNC2H1* identified by WES. The discovery of new clinical manifestations and genetic variants may facilitate the prenatal diagnosis of skeletal dysplasia. The possible causes of the affected fetuses in four families were preliminarily identified from the perspective of genetics, which provided strong evidence for their clinical diagnosis and reliable molecular basis for genetic counseling and prenatal diagnosis.

## Materials and methods

### Patients

The present study was approved by the ethics committee of Maternal and Child HealthCare Hospital of Shandong Province, and informed consent was acquired from each parent of the fetuses. Between May 2019 to April 2022, we collected 4 fetuses with skeletal dysplasia on ultrasound and with *DYNC2H1* mutation. The parents and other family numbers were in normal physical condition, and denied any adverse history. The maternal age range at diagnosis of abnormal bone was 23–33 years, and the gestation age range at diagnosis was 14–31 weeks. Multidisciplinary consultation including genetic counseling regarding the risk of abnormal bone and the chance of surgery as well as the benefits and limitations of WES were introduced to the couples.

### WES analysis

Fetal samples of case 1 and case 4 were from amniotic fluid during the invasive prenatal diagnosis. Fetal samples of case 2 and case 3 were from muscle tissue of the aborted fetus. The peripheral blood was collected in EDTA-containing tubes from the four couples. Proband-WES sequencing was performed in family 2, and Trio-WES sequencing was selected in the other three families. WES sequencing was tested by Yin Feng Gene Technology Co., Ltd. (Jinan, China). Briefly, genomic DNA was extracted from peripheral blood for each sample using Magnetic Universal Genomic DNA Kit (TIANGEN, China). The quality and quantity of each DNA sample were detected by 1% agarose gel electrophoresis, NanoPhotometer (IMPLEN, CA, United States) and Qubit^®^ 3.0 Flurometer (Life Technologies, CA, United States). Then, DNA libraries were prepared using Illumina standard protocol. Based on the manufacturer’s instructions, the exome was captured using IDT xGen Exome Research Panel v1.0 (Integrated DNA Technologies, Coralville, Iowa, United States), and sequenced using Illumina Novaseq 6,000 platform (Illumina Inc., San Diego, CA, United States) with a depth of 100-fold. The data obtained by WES sequencing was then used for mutation hazard prediction, genotype-phenotype correlation analysis and mutation screening.

### Sanger sequencing

In order to validate the identified mutation of *DYNC2H1* by whole exome sequencing, we performed Sanger sequencing in fetuses and their parents. DNA was extracted using TIANGEN Universal DNA Purification kit (TIANGEN), and PCR was carried out with PCR instrument. The product was sequenced by Yin Feng Gene Technology Co., Ltd. using ABI 3730xl DNA Analyzer (ABI, United States).

### Bioinformatics analysis

After sequencing, bcl2fastq software (Illumina) was performed for base-call file conversion and emultiplexing. The resulting fastq data was analyzed by internal quality control software to remove low quality reads. The low quality reads were filtered out from all sequencing dates using a quality score 20 (Q20), and then burrows Wheeler comparator (BWA) software was performed to align to human reference genome (hg37), Picard tools were used to mark the duplicated reads. Single nucleotide variants (SNVs) and indels identification were carried out using GATK software, and ANNOVAR software was used to annotate the detected mutation sites. SNVs/Indels were compared with the dbSNP (https://www.ncbi.nlm.nih.gov/snp), 1000 Genomes Project (1000 GP) (https://browser/1000genomes.org/), Exome Aggregation Consortium (https://exac.broadinstitute.org/) and Exome Variant Server databases (https://evs.gs.washington.edu/EVS). Databases such as OMIM (https://www.omim.org), ClinVar (https://www.ncbi.nlm.nih.gov/clinvar), Human Gene Mutation Database (https://www.hgmd.org) and SwissVar (https://www.bioinfo.org/wiki/index.php/SwissVar) were used to determine mutation harmfulness and pathogenicity where appropriate. All whole-exome variants were subjected to biological effects analysis, which included the use of programs such as SIFT (https://sift.jcvi.org), MutationTaster (https://www.mutationtaster.org), PolyPhen-2 (https://genetics.bwh.harvard.edu/pph2), PROVEAN (https://provean.jcvi.org/index.php), SpliceAI (https://spliceailookup.broadinstitute.org/#) and MaxEntScan (https://genes.mit.edu/burgelab/maxent/Xmaxentscan_scoreseq.html) to predict whether an amino acid substitution or indel has an important biological effect. Variants were selected after combining the clinical information, and then pathogenicity was determined according to the American College of Medical Genetics and Genomics (ACMG) guidelines. The detailed variant calling, annotating and filtering strategy is described in Supplementary Figure S1.

### Minigene splicing assay

The minigene splicing assay was used to study the effect of the c.8833-1G>A variant on mRNA splicing. In brief, the *DYNC2H1* wild-type (WT) and *DYNC2H1*: c.8833-1G>A mutant (MT) minigene plasmids were established using two vector including pcMINI and pcMINI-C (Bioeagle, China). Then, the WT and MT forms of minigene minigene constructs were confirmed using sanger sequencing. Besides, HeLa and HEK293T cells were transfected into these plasmids for 48 h, and the RNA sample was extracted and reversely transcripted into cDNA. Ultimately, the effect of the c.8833-1G>A variant on mRNA splicing was assessed by PCR and sanger sequencing.

### Protein-protein interaction network and gene ontology (GO) analysis

STRING (https://string-db.org/) was utilized to establish protein-protein interaction network associated with *DYNC2H1*. The GO analysis of these genes in protein-protein interaction network was downloaded in STRING and was generated by https://www.bioinformatics.com.cn, a free online platform for data analysis and visualization.

## Results

### Patient and sonographic findings

**FIGURE 1:**
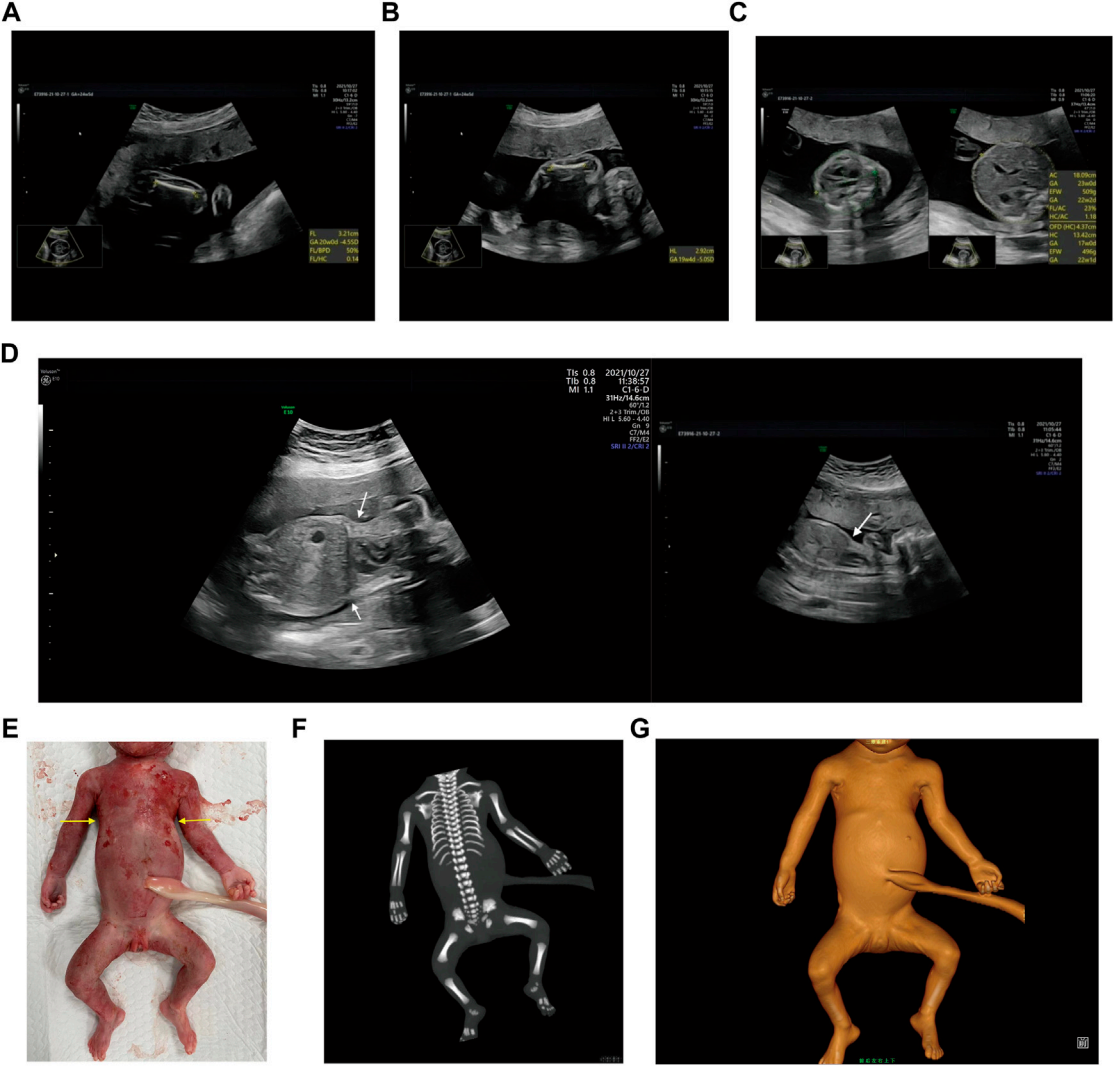
Antenatal ultrasound, photos and radiographs of the foetus 1 **(A)** The femur length was 3.2 cm. **(B)** The humerus length was 2.9 cm. **(C)**The thoracic circumference and abdominal circumference was 13.4 and 18.1 cm respectively. **(D)** Sagittal view of fetal thorax and abdomen showed a “thoracoabdominal notch” in the junction of thorax and abdomen (indicated by the arrows). **(E)** Appearance of the foetus after induced labor. A narrow thorax was observed. **(F, G)** Radiographs and CT 3-dimensional image reconstruction of the foetus. A narrow thorax and short long bones were observed.

Case 1: A routine ultrasound at 24 weeks of gestation observed multiple anomalies of the fetus, including short long bones and narrow thorax (Figures 1A–D). Detailed ultrasound results were shown in Table 1. Amniocentesis was performed to extract amniotic fluid, and then Trio WES sequencing was carried out. Ultimately, the parents decided to terminate the pregnancy at 27 weeks of gestation. We tracked the clinical phenotype of the induced fetus (Figure 1E), a narrow thorax was observed. X-ray CT scan examination was executed, and verified that the fetus presented narrow thorax and short long bones (Figures 1F, G). Prior to the proband, the mother had one history of adverse pregnant: At 24 weeks of gestation, pregnancy was terminated due to the discovery of the same ultrasonic detection patterns (Femurlength (FL):3.3 cm (−3.988SD), Humerus length (HL):3.2 cm(−3.903SD), with a normal HC (22.1 cm) and AC (19.1 cm) for the gestational age) and no related genetic analysis was executed.

**TABLE 1:** Clinical details: antenatal ultrasound and molecular studies of cases.

Features gestational age of diagnosis	Case 1	Case 2	Case 3	Case 4
	24 weeks 5 days	31 weeks	14 weeks	25 weeks 2 days
	Measurement (SD)	Measurement (SD)	Measurement (SD)	Measurement (SD)
HC	23.1 cm	30.8 cm	9.4 cm	21.9 cm
BPD	6.5 cm	8.8 cm	2.6 cm	5.9 cm
FL	3.2 cm (−4.5SD)	4.4 cm	0.45 cm	3.7 cm
HL	2.9 cm (−5.0SD)	4.2 cm	0.65 cm	21.9 cm
Foot length	4.6 cm	NA	NA	4.6 cm
Thoracic circumference ThC (cm)	13.4 cm	NA	NA	NA
Femur foot ratio	0.69	NA	NA	0.8
AC	18.1 cm	38 cm	7.7 cm	19 cm
ThC/AC	0.74	NA	NA	NA
Clinical phenotype	Short long bones, short ribs, narrow chest	Short long bones, Seroperitoneum, polyhydramnios	Severe short long bones deformity, hand and foot posture abnormalities, cardiac abnormalities, single umbilical artery	Short femur length and slightly bending
Parental consanguinity	Determined	Determined	Determined	Determined
*DYNC2H1* mutation	Compound heterozygous c.3842A>C (p.Tyr1281Ser) and c.8833-1G>A	Compound heterozygous c.8617A>G (p.Met2873Val) and c.7053_7054del (p.Cys2351Ter)	Compound heterozygous c.5984C>T (p.Ala1995Val) and c.10219C>T (p.Arg3407Ter)	Compound heterozygous c.5256del (p.Ala1753GlnfsTer13) and c.9737C>T (p.Thr3246Ile)

Abbreviations: AC, abdominal circumference; BPD, biparietal diameter; FL, femur length; HC, head circumference; HL, humerus length; NA, not available; SD, standard deviation; ThC, thoracic circumference.

**FIGURE 2:**
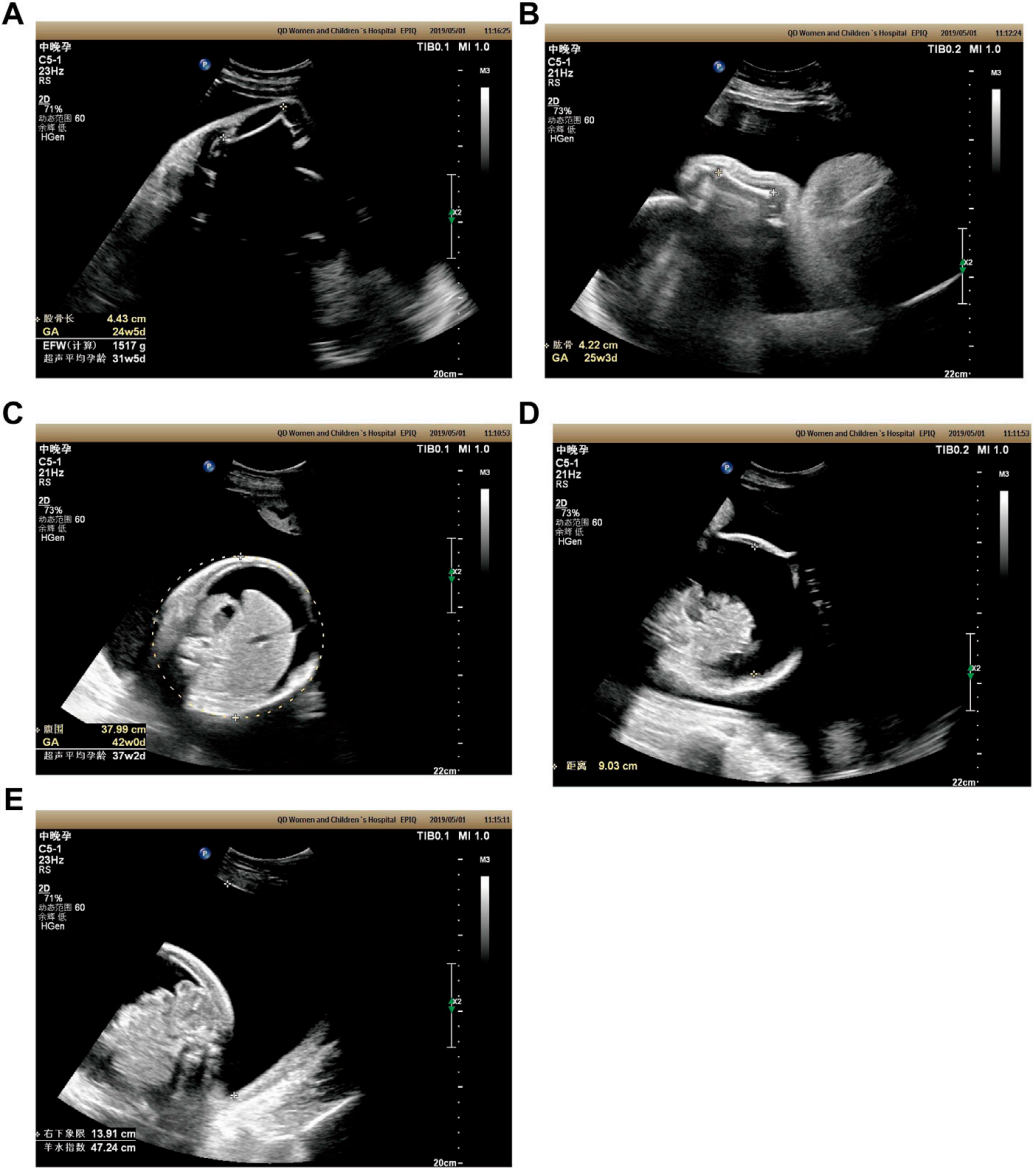
Antenatal ultrasound of foetus 2 **(A)** The femur length was 4.4 cm. **(B)** The humerus length was 4.2 cm. **(C)**The abdominal circumference was 38.0 cm. **(D)** The seroperitoneum was 9.0 cm. **(E)**The amniotic fluid index was 47.0 cm.

Case 2: At 31 weeks of gestation, the ultrasound results displayed short long bones, seroperitoneum and polyhydramnios (Figures 2A–E). More detailed ultrasound results were shown in Table 1. The parents were fully informed of fetal abnormality. The parents strongly requested the induction of labor. With the approvement of the institutional ethics committee of Shandong Provincial Maternal and Child HealthCare Hospital, the pregnancy was terminated for fetal anomalies. The parents decided to conduct the proband WES analysis.

**FIGURE 3:**
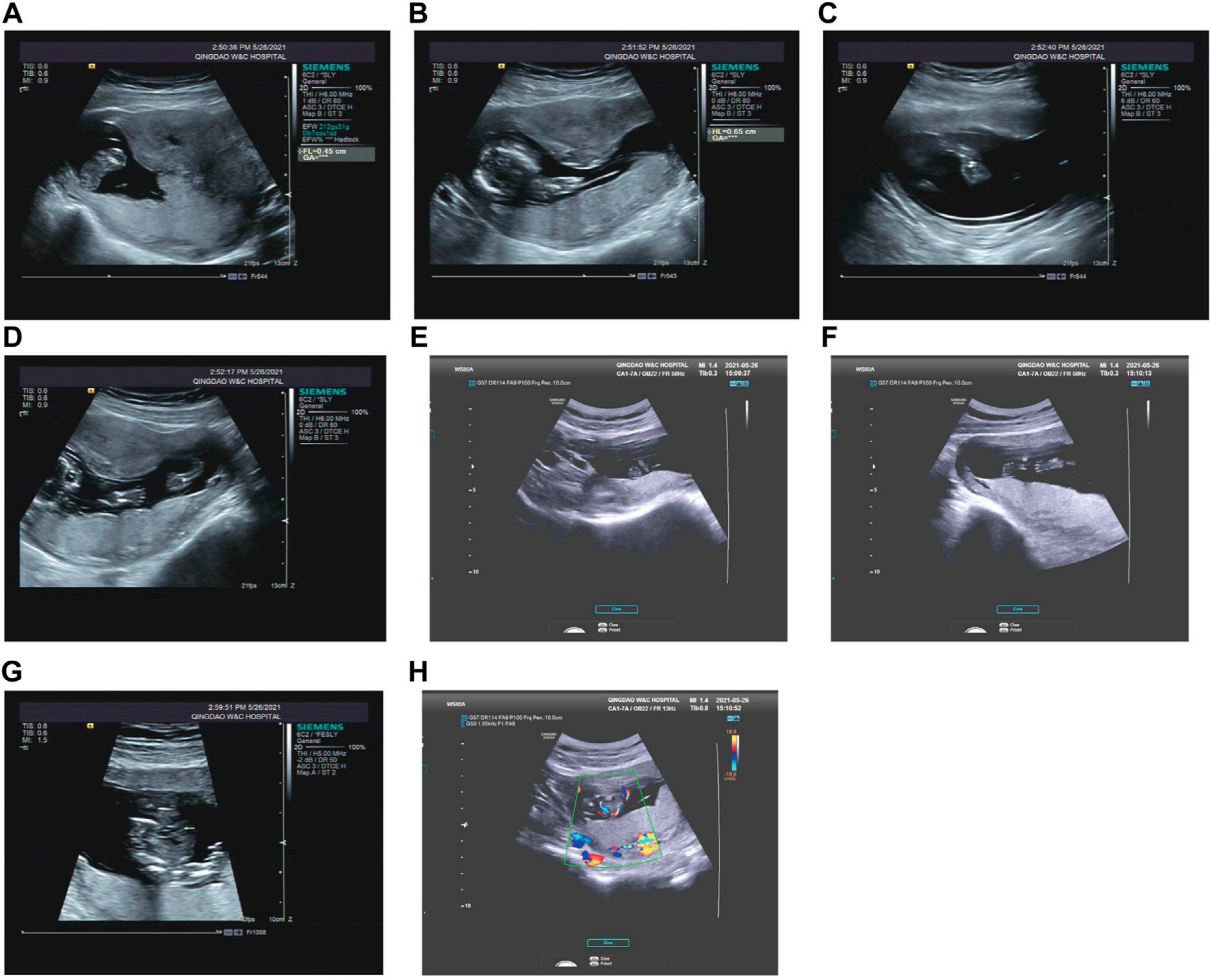
Antenatal ultrasound of foetus 3 **(A)** The femur length was 0.45 cm. **(B)** The humerus length was 0.65 cm. **(C)** The ulna and radius were not clearly shown. **(D)** The tibiofibula was not clearly visualized. **(E)** Polydactyly of both feet. **(F)** Posture abnormalities of both feet. **(G)** Abnormal structure of four-chamber heart. **(H)** single umbilical artery.

Case 3: At 14 weeks of gestation, the ultrasound results showed severe short limbs, abnormal posture of hand and foot, cardiac malformation and single umbilical artery (Figures 3A–H. More detailed ultrasound results were shown in Table 1. Fetus 3’s parents had a strong desire to induce labor. After approval by the institutional ethics committee of Shandong Provincial Maternal and Child Healthcare Hospital, the pregnancy was terminated due to fetal abnormality. The parents decided to terminate the pregnancy and undergo Trio WES analysis.

**FIGURE 4:**
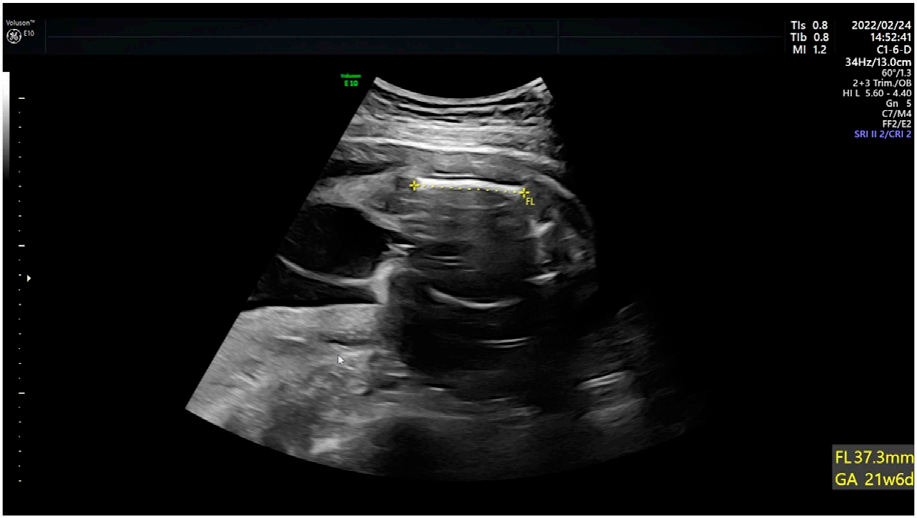
Antenatal ultrasound of foetus 4 The femur length was 3.7 cm and slightly bowing.

Case 4: Ultrasound of this fetus at 25 weeks of gestation showed short femur length and slightly bending (Figure 4). More detailed ultrasound results were shown in Table 1. Amniocentesis was performed to extract amniotic fluid, and then Trio WES sequencing was carried out.

### WES analysis

**FIGURE 5:**
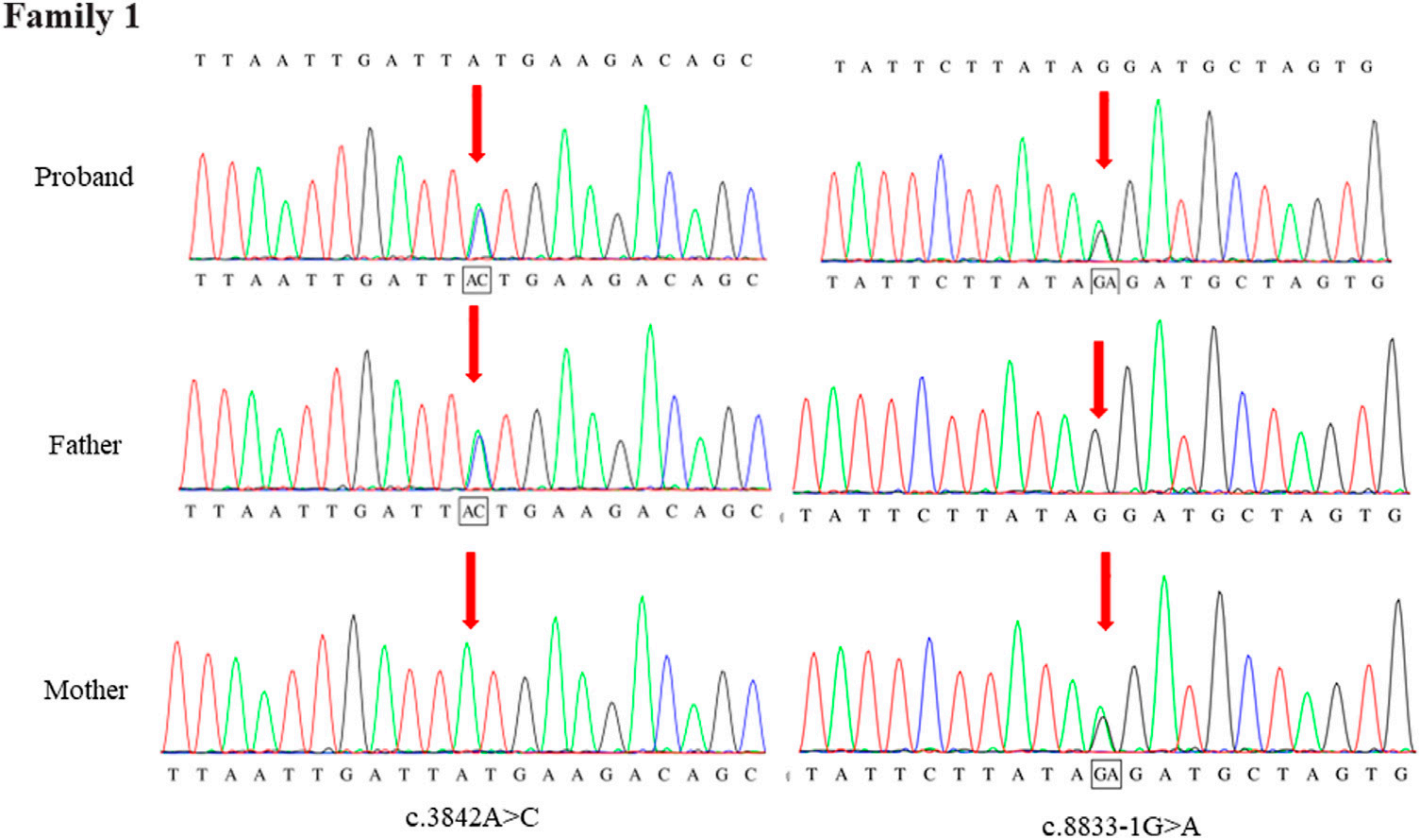
Sanger sequence analysis of the mutations of *DYNC2H1* gene in the family 1.

**FIGURE 6:**
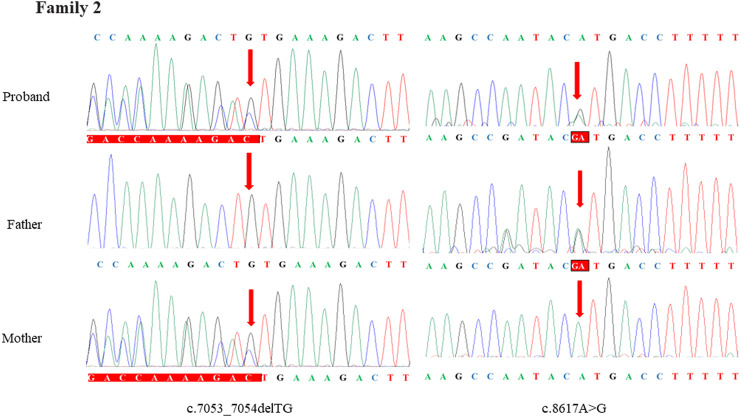
Sanger sequence analysis of the mutations of *DYNC2H1* gene in the family 2.

**FIGURE 7:**
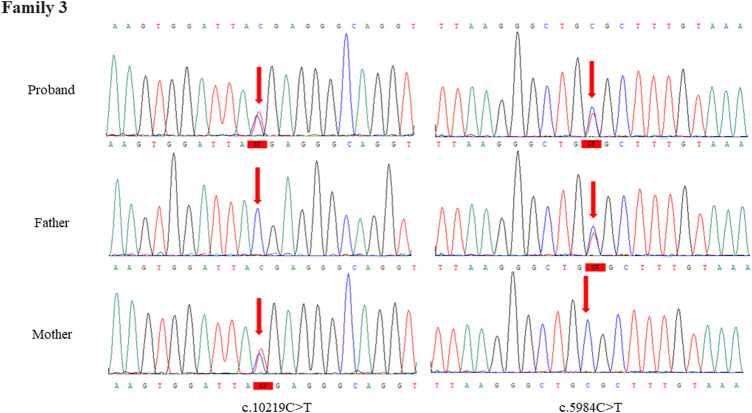
Sanger sequence analysis of the mutations of *DYNC2H1* gene in the family 3.

**FIGURE 8:**
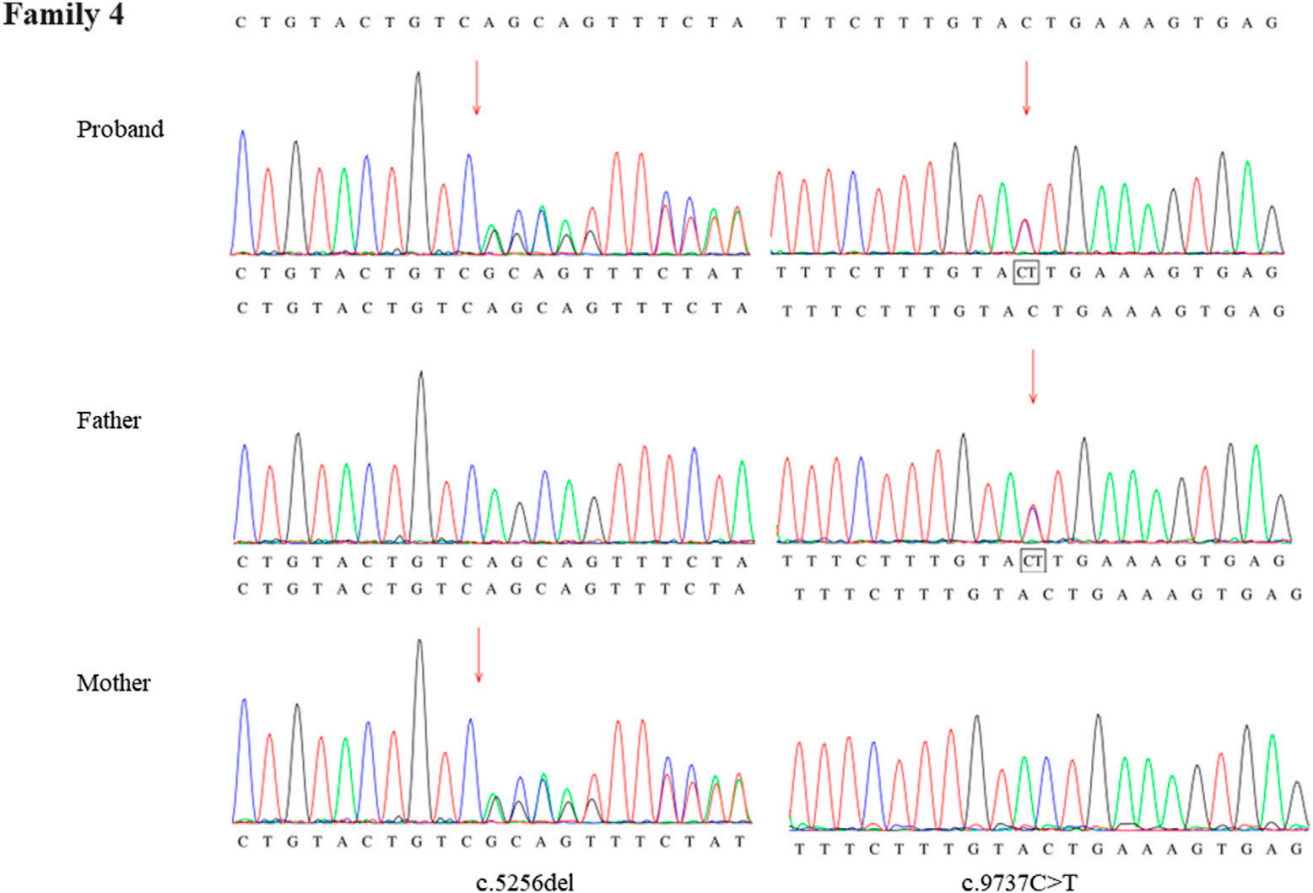
Sanger sequence analysis of the mutations of *DYNC2H1* gene in the family 4.

**FIGURE 9:**
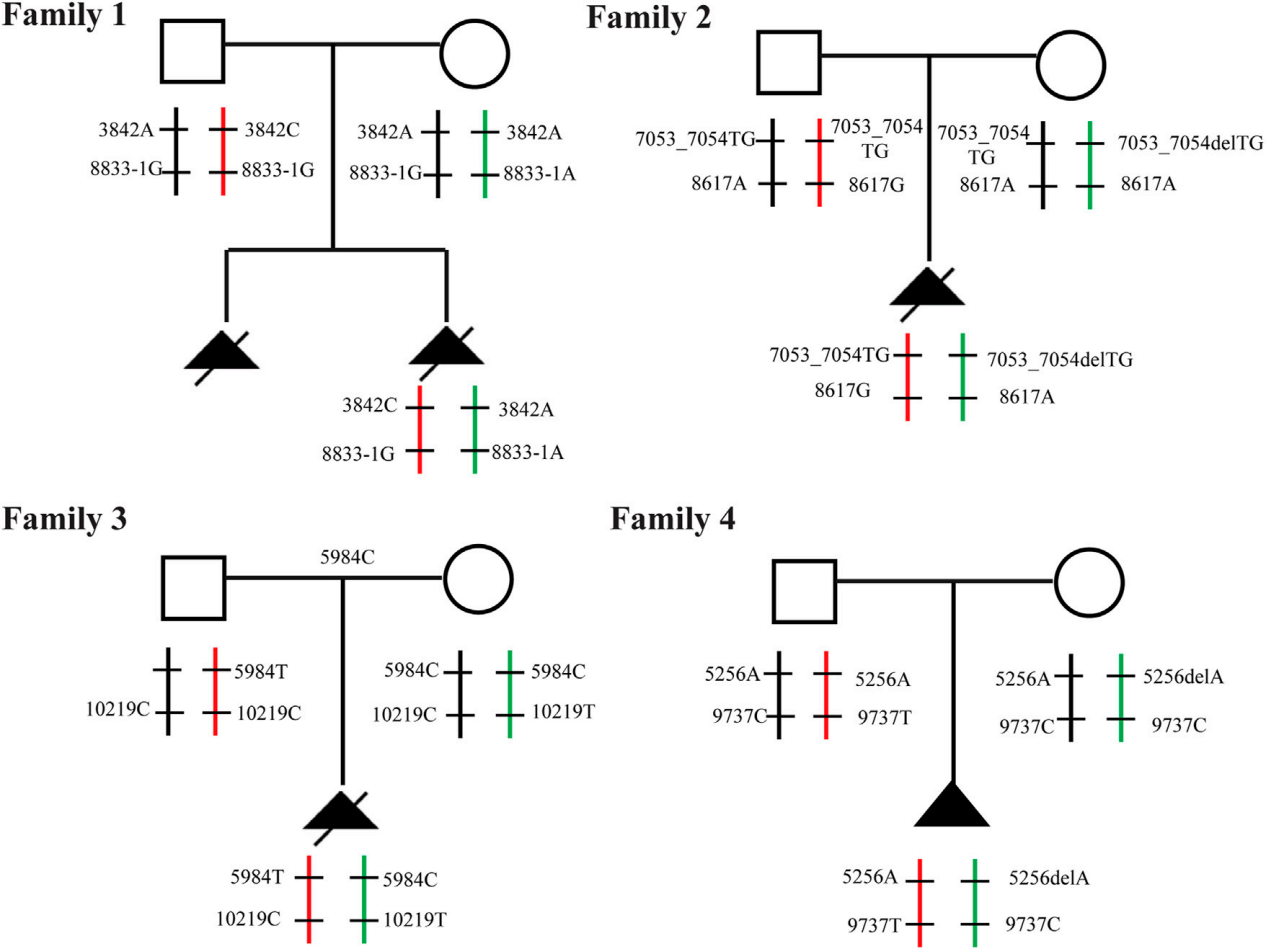
Familial pedigree Pedigree with autosomal recessive SRTD3 displaying cosegregation of the compound heterozygous changes within the *DYNC2H1* gene in four families.

Through the bioinformatics analysis, two compound heterozygous variants in *DYNC2H1* (NM_001080463.2), c.3842A>C (p.Tyr1281Ser) and c.8833-1G>A, were identified as novel mutations for the fetus 1 (Table 1). Among which, the father carried c.3842A>C (p.Tyr1281Ser), and mother had c.8833-1G>A (Figure 5). Sanger sequencing was also used to prove the variants in the family 1. Following the American College of Medical Genetics and Genomics (ACMG) guidelines, both the c.3842A>C (p.Tyr1281Ser) and c.8833-1G>A in *DYNC2H1* gene were predicted to be variants of uncertain significance mutations (Table 2). In fetus 2, WES also identified a compound heterozygous variation in the *DYNC2H1* gene, comprising of two variant, namely, c.8617A>G (p.Met2873Val) and c.7053_7054del (p.Cys2351Ter) (Table 1). Sanger sequencing was applied to confirm two mutations in family 2 and found c.8617A>G (p.Met2873Val) mutation was inherited from father and c.7053_7054del (p.Cys2351Ter) was inherited from mother (Figure 6). According to the ACMG guidelines, c.8617A>G (p.Met2873Val) in *DYNC2H1* gene was classified as uncertain significance mutations, and c.7053_7054del (p.Cys2351Ter) was classified as pathogenic (PVS1+PM2_Supporting + PM3) (Table 2). Two compound heterozygous mutations c.5984C>T (p.Ala1995Val) and c.10219C>T (p.Arg3407Ter) in the *DYNC2H1* gene were observed by WES from fetus 3. Of which, the c.5984C>T (p.Ala1995Val) mutation was inherited from father and c.10219C>T (p.Arg3407Ter) was inherited from mother. Sanger sequencing also verified that the c.5984C>T (p.Ala1995Val) mutation was inherited from father and c.10219C>T (p.Arg3407Ter) was inherited from mother (Figure 7). Based on the ACMG guidelines, both the c.10219C>T (p.Arg3407Ter) and c.10219C>T (p.Arg3407Ter) in *DYNC2H1* gene were classified as pathogenic and the evidence their used was PM3_Strong + PM1+PM2_Supporting + PM5 and PVS1+PM2_Supporting + PM3, respectively (Table 2). Two novel compound heterozygous mutations of *DYNC2H1*, c.5256del (p.Ala1753GlnfsTer13) and c.9737C>T (p.Thr3246Ile), were detected in fetus 4, which were inherited from father and mother, respectively. Sanger sequencing also verified that the c.5256del (p.Ala1753GlnfsTer13) mutation was inherited from father and c.9737C>T (p.Thr3246Ile) was inherited from mother (Figure 8). Following the ACMG guidelines, the c.5256del (p.Ala1753GlnfsTer13) variant was rated as likely pathogenic (PVS1+PM2_Supporting), whereas c.9737C>T (p.Thr3246Ile) variant was rated as uncertain significance (Table 2). The pedigree diagram of the four families is shown in Figure 9. In addition, no rare variants were found in any of the other genes associated with skeletal dysplasia in these four patients. Whole-exome data using VCF files in the Integrated Genomics Viewer (IGV) depicting the genotypic information of *DYNC2H1* candidate variant in all samples (Supplementary Figures S2–S5). Moreover, a detailed list of variants identified by whole-exome sequencing in all cases is shown in Supplementary Tables S1–S4.

**TABLE 2:** Pathogenicity analysis of the *DYNC2H1* variants and frequency in the normal population.

Case	Location	Nucleotide change	Amino acid change	Prediction of biological hazard			Normal population databas			ACMG
				PolyPhen-2	SIFT	MutationTaster	ESP	1000Genome	ExAC	
1	chr11:103,027,214	c.3842A>C	p.Tyr1281Ser	Possibly damaging	Tolerated	Disease causing	Not available	Not available	Not available	Uncertain significance PM2_Supporting + PM3
	chr11:103,090,643	c.8833-1G>A		Not available	Not available	Disease causing	Not available	Not available	Not available	Likely pathogenic PS3+PVS1_S + PM2_Supporting
2	Chr11:103,082,595	c.8617A>G	p.Met2873Val	Benign	Tolerated	Disease causing	Not available	Not available	Not available	Uncertain significance
	chr11:103,058,226–10305 8,227	c.7053_7054del	p.Cys2351Ter	Not available	Not available	Not available	Not available	Not available	Not available	Pathogenic PVS1+PM2_Supporting + PM3
3	chr11:103,048,394	c.5984C>T	p.Ala1995Val	Possibly damaging	Deleterious	Disease causing	Not available	Not available	Not available	Pathogenic PM3_Strong + PM1+PM2_Supporting + PM5
	chr11:103,124,169	c.10219C>T	p.Arg3407Ter	Not available	Not available	Disease causing	Not available	Not available	Not available	Pathogenic PVS1+PM2_Supporting + PM3
4	chr11:103,041,719	c.5256del	p.Ala1753GlnfsTer13	Not available	Not available	Not available	Not available	Not available	Not available	Likely pathogenic PVS1+PM2_Supporting
	chr11:10,310 186	c.9737C>T	p.Thr3246Ile	Possibly damaging	Deleterious	Disease causing	Not available	Not available	Not available	Uncertain significance PM2_Supporting + PM3

### Mutation analysis

**FIGURE 10:**
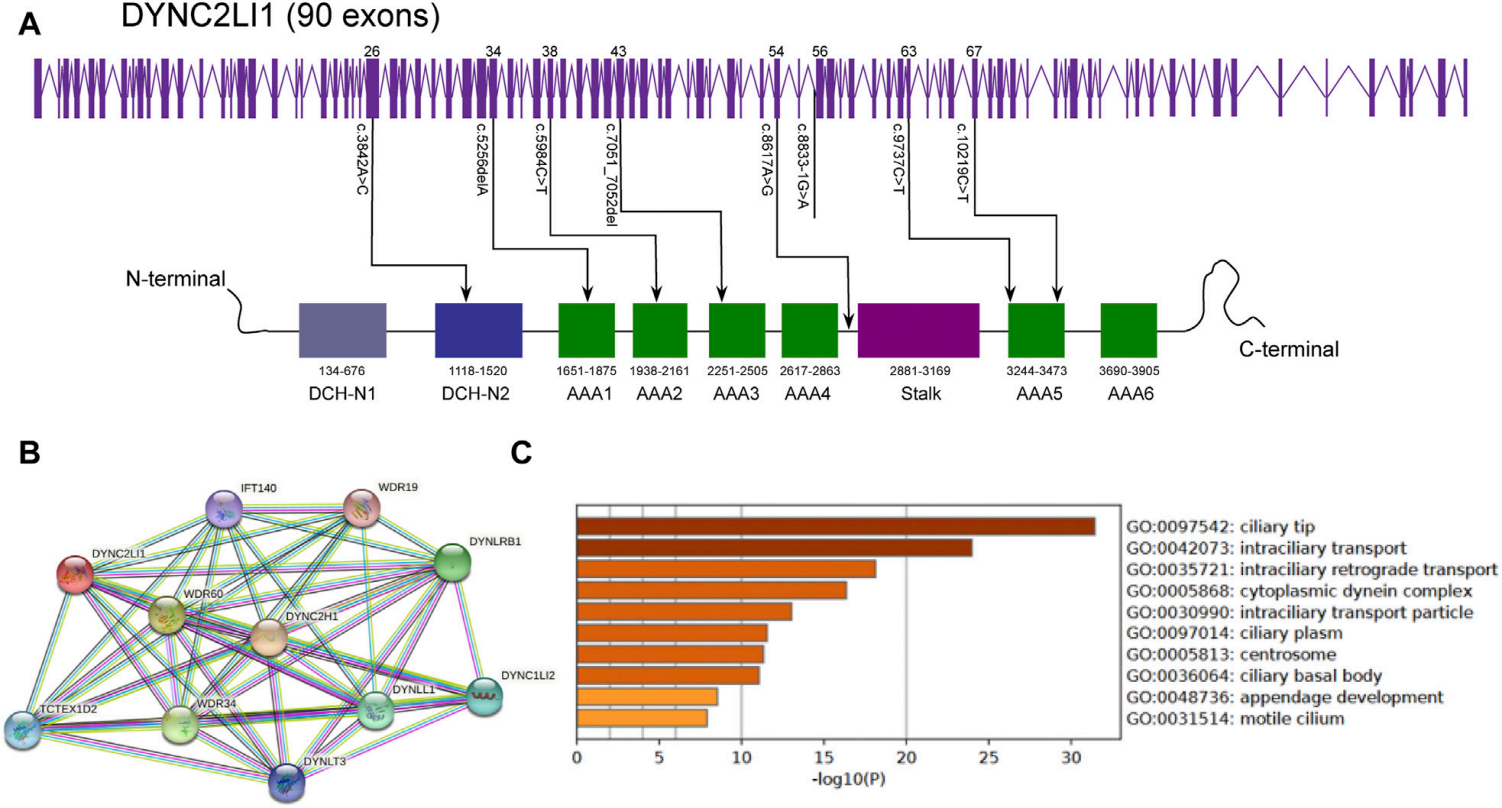
*DYNC2H1* gene and peptide chain schematics, the protein-protein interaction network and GO terms of genes associated with *DYNC2H1* **(A)** The specific positions of these eight variants in the *DYNC2H1* gene and peptide chain schematics. **(B)** The protein-protein interaction network associated with *DYNC2H1*. Circles are used to represent nodes, and lines are used to represent edges. **(C)** GO terms of these genes in protein-protein interaction network. The X-axis represents the -log 10(P) and the Y-axis represents the GO term.

Figure 10A depicted the specific positions of these eight variants in the *DYNC2H1* gene and peptide chain schematics. The c.3842A>C (p.Tyr1281Ser), c.5984C>T (p.Ala1995Val), c.7053_7054del (p.Cys2351Ter)variants of *DYNC2H1* were located in DHC_N2 region, AAA1domain, AAA2 domain and AAA3 domain, respectively. The total of two *DYNC2H1* variants including c.9737C>T (p.Thr3246Ile) and c.10219C>T (p.Arg3407Ter) were located in the AAA5 domain. In addition, these eight mutations were predicted using the ONLINE pathogenesis prediction programs including PolyPhen-2, SIFT and MutationTaster (Table 2). Furthermore, eight variants were not found in the control population including ESP, the 1,000 Genomes database, and EXAC databases (Table 2). Based on these results, we speculate that these variants of *DYNC2H1* are responsible for the pathogenesis of SRTD3.

### Protein-protein interaction network and functional annotation analysis

The protein-protein interaction network associated with *DYNC2H*1 was produced by STRING. The protein-protein interaction network was consisted of 11 nodes and 51 edges (Figure 10B). Among which, the top one protein with higher degrees was *DYNC2H1* (degree = 10). Then, these 11 genes in protein-protein interaction network were utilized to perform the GO enrichment analysis. Top 10 significantly GO terms were displayed in the Figure 10C. GO terms enrichment analysis showed that ciliary tip, intraciliary transport, intraciliary retrograde transport, cytoplasmic dynein complex, intraciliary transport particle, ciliary plasm, centrosome, ciliary basal body, appendage development, motile cilium were significantly enriched GO terms.

### *In vitro* minigene splice assay

**FIGURE 11:**
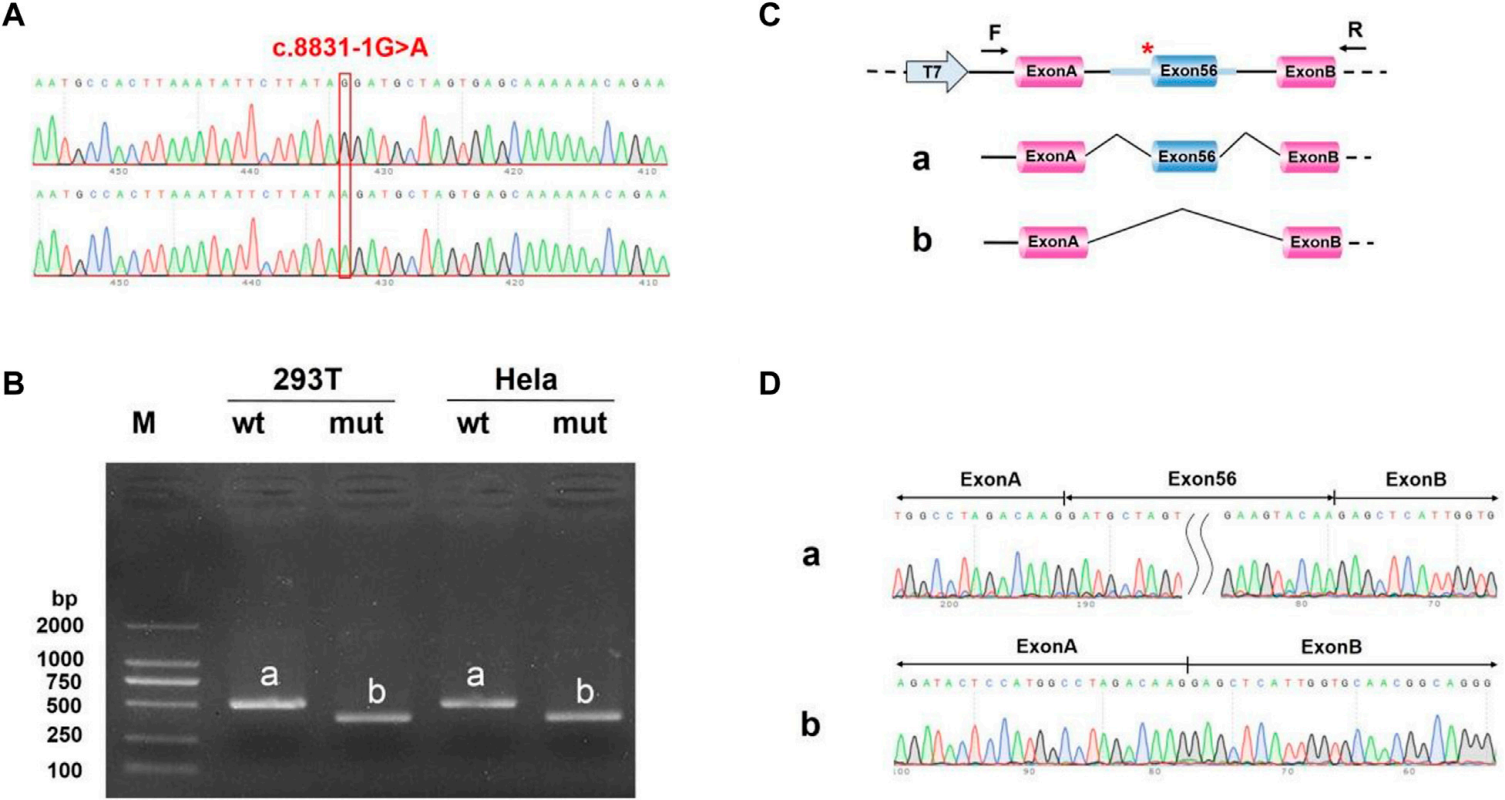
Minigene analysis based on pcMINI-*DYNC2H1*-wt/mut recombinant vector **(A)** Sanger sequencing results of the recombinant vector. **(B)** Electrophoresis results of transcript PCR products in both 293T and HeLa cell lines. **(C)** A schematic splicing mechanism of the *DYNC2H1* c.8833-1G > A variant. **(D)** Sanger sequencing of PCR products. Red * indicates the mutation site.

**FIGURE 12:**
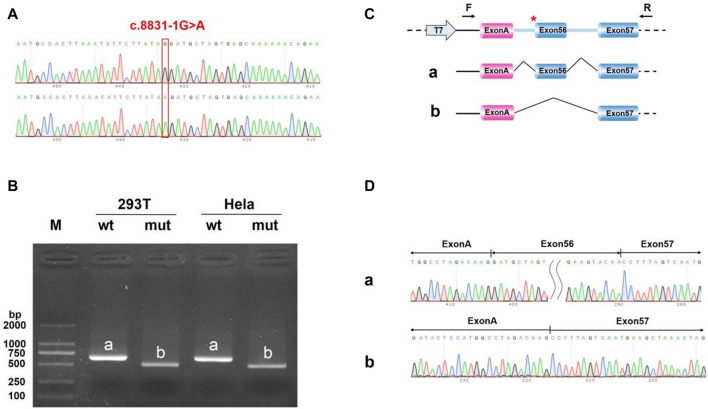
Minigene analysis based on pcMINI-C-*DYNC2H1*-wt/mut recombinant vector **(A)** Sanger sequencing results of the recombinant vector. **(B)** Electrophoresis results of transcript PCR products in both 293T and HeLa cell lines. **(C)** A schematic splicing mechanism of the *DYNC2H1* c.8833-1G > A variant. **(D)** Sanger sequencing of PCR products. Red * indicates the mutation site.

We uncovered the impact of c.8833-1G > A on splicing according to the vitro minigene splicing assay. The results of sanger sequencing showed that both wild-type and mutant Minigene were successfully inserted into the corresponding vectors (Figures 11A, 12A). As shown in Figures 10B, 11B, the minigene splice assay results indicated that the MT plasmid yielded a smaller transcript compared with the wild plasmids. Therefore, the abnormal splicing pattern caused by the c.8833-1G > A variant is inferred as displayed in Figures 11C, 12C. In addition, sanger sequencing was determined this result (Figures 11D, 12D). These results showed that the mutation c.8833-1G > A would affect the normal splicing of gene mRNA, and the detection results of pcMINI and PCMINI-C were consistent, both of which led to the jump of exon 56. Collectively, above results indicated that the c.8833-1G > A was a splicing variant causing exon 56 skipping.

## Discussion

Despite the emergence of skeletal dysplasia in each individual is relatively rare, skeletal dysplasia accounts for a considerable number of congenital malformations in neonates (Lord et al., 2019; He et al., 2021). About 40% of skeletal dysplasia manifests at birth or before birth, and ultrasound remains an essential first-line screening way (Mary et al., 2019; Tang et al., 2020). Due to the diversity and variability of skeletal dysplasia manifestations and their widely overlapping phenotypes, ultrasound alone cannot effectively distinguish between various skeletal dysplasia. It has been reported that prenatal WES can help increase the detection rate of fetal skeletal abnormalities (Lord et al., 2019; Zhao et al., 2021). Therefore, more accurate gene diagnosis is of great value for obstetricians to evaluate fetal prognosis, provide genetic counseling and determine the optimal treatment. In the current study, by using trio-WES and proband-WES study with comprehensive analysis, eight mutations (4 pairs of compound heterozygous sites) of *DYNC2H1* were identified in four Chinese families with fetal skeletal dysplasia, supporting a diagnosis of SRTD3 (OMIM; 613091).

At present, approximately twenty genes have been found to be involved in SRTD3 (Badiner et al., 2017). Among them, *DYNC2H1* gene is more common and has been identified as the causative gene of SRTD3. *DYNC2H1*, located on chromosome 11q22.3 and contained 90 exons, is a cytoplasmic dynein involved in the retrograde transport of cilia, which is an important component of endochondral bone development (Vig et al., 2020; Zhang et al., 2020). Fetuses with SRTD3 usually displays with extremely shortened long bones, round metaphyseal ends and lateral spikes, a small and narrow thorax, and preaxial and postaxial polydactyly, sometimes exhibiting with bowing femora, genitals and multiple deformities. In our study, four affected fetuses showed different SRTD3 phenotypes. SRTD3 disease has been found to have clinical and genetic heterogeneity, such as significant differences in the course and severity of the thoracic phenotype between individuals with different alleles, indicating that the *DYNC2H1* phenotype may be affected by allele modification (Zhang et al., 2020).

Currently, a large number of different variants of *DYNC2H1* gene have been reported in many databases, but there are no hot spot variants. Most of these variants are compound heterozygous variants, generally one is missense variant, and the other is frameshift or truncated protein variant (Chen et al., 2018; Thakur et al., 2021; Xia et al., 2021). To the best of our knowledge, only four were homozygous mutations of *DYNC2H1* gene in all cases (Geng et al., 2020). The low reported rate of homozygous mutations in *DYNC2H1* may be due to the early death of homozygous embryos. The *DYNC2H1* compound heterozygous variant fits the autosomal recessive inheritance pattern of SRTD3. Cheng et al. report a Chinese foetus with SRTD3 carrying a c.11483T>G and c.2106+3A > T mutations who exhibited severe SRTD3 phenotypes (Cheng et al., 2022). Zhang et al. identify two novel compound heterozygous variants c.358G>T and c.928A>T in *DYNC2H1* in two Chinese fetus, but the phenotypes of the two fetuses showed different SRTD3 phenotypes (Zhang et al., 2020). Deng et al. find two novel compound heterozygous mutations c.2992C>T and c.12836G>C of the *DYNC2H1* gene in a fetus with polyhydramnios in China (Deng et al., 2018). In our study, WES also identified a compound heterozygous variation c.8617A>G (p.Met2873Val) and c.7053_7054del (p.Cys2351Ter) of *DYNC2H1* gene in fetus 2, which also presented with polyhydramnios. Therefore, we hypothesized that polyhydramnios may also be one of the prenatal manifestations of SRTD3 fetuses.

In our study, eight mutations, 4 pairs of compound heterozygous variants including c.3842A>C (p.Tyr1281Ser) and c.8833-1G>A, c.8617A>G (p.Met2873Val) and c.7053_7054del (p.Cys2351Ter), c.5984C>T (p.Ala1995Val) and c.10219C>T (p.Arg3407Ter), c.5256del (p.Ala1753GlnfsTer13) and c.9737C>T (p.Thr3246Ile), were found in four Chinese fetus. According to the ACMG guidelines, c.8617A>G (p.Met2873Val), c.7053_7054del (p.Cys2351Ter), c.5984C>T (p.Ala1995Val), c.10219C>T (p.Arg3407Ter) and c.5256del (p.Ala1753GlnfsTer13) were rated as pathogenic or likely pathogenic variants, others variants were predicted to be variants of uncertain significance mutations. Four of the eight variants, including c.3842A>C (p.Tyr1281Ser), c.8833-1G>A, c.7053_7054del (p.Cys2351Ter), c.5256del (p.Ala1753GlnfsTer13), were not reported in HGMD and ClinVar databases. Among which, c.10219C>T (p.Arg3407Ter), c.5984C>T (p.Ala1995Val) and c.9737C>T (p.Thr3246Ile) were reported in ClinVar databases, and c.8617A>G (p.Met2873Val), c.10219C>T (p.Arg3407Ter), c.5984C>T (p.Ala1995Val) were found in HGMD databases. Herein, we reported two novel compound heterozygous variants c.3842A>C (p.Tyr1281Ser) and c.8833-1G>Ain *DYNC2H1* in fetus 1. Moreover, c.7053_7054del (p.Cys2351Ter) and c.5256del (p.Ala1753GlnfsTer13) were novel variants found in fetus 2 and fetus 4, respectively. In addition, the parents in all cases were without any minor symptoms. Moreover, parental consanguinity is presented in each case. As a whole, the discovery of these novel mutations not only enriched the mutation database, but also provided a basis for genetic counseling and further prenatal diagnosis of families.

SRTD3 has been reported to have a variety of phenotypes such as shortened limbs, a narrow trunk, and associated visceral abnormalities with or without polydactyly and so on. Chen et al. report a Chinese fetus with SRTD3 carrying c.8077G > T and c.11741_11742delTT mutations in *DYNC2H1* who exhibited short limbs, a narrow chest and bilateral polydactyly of the hands and feet phenotypes (Chen et al., 2018). Cheng et al. identify novel compound variants of *DYNC2H1* (c.11483T > G and c.2106 + 3A > T), which presented with abnormal rib curvature, narrow thorax, bilateral hypoplastic lungs, bilateral polydactyly, syndactyly, and foetal visceral situs inversus with mirror-image dextrocardia (Cheng et al., 2022). Xia et al. report novel compound heterozygous variants of *DYNC2H1* (c.6591_6593delTGG and c.7883T>C) in a fetus, which was exhibited a long, narrow thorax with short ribs, shortened long bones, spurs at the metaphysis of the long bones and congenital bowing of the femurs (Xia et al., 2021). Mei et al. find compound heterozygous mutations of *DYNC2H1* gene (c.1151 C>T and c.4351 C>T) in the fetus with SRTD3, which were showed short limbs, a narrow thorax, short ribs with marginal spurs, and polydactyly (Mei et al., 2015). He et al. find a Chinese fetus carrying compound heterozygous variants of *DYNC2H1* gene (c.2225T > G and c.10219C > T) in the fetus with phenotypes including an increased nuchal translucency, a narrow thorax, severely short limbs, postaxial polydactyly of right hand, endocardial cushion defect, megacystis and omphalocele (He et al., 2020). In our study, two compound heterozygous mutations (c.5984C>T (p.Ala1995Val) and c.10219C>T (p.Arg3407Ter)) in the *DYNC2H1* gene were observed by WES from fetus 3, and fetus 3 phenotypes containing severe short limbs, abnormal posture of hand and foot, cardiac malformation and single umbilical artery. Both the case reported by He et al. and case 3 in this study carried the c.10219C>T (p.Arg3407Ter) mutation and both fetuses had severely short limb and cardiac malformations. The c.10219C>T (p.Arg3407Ter) mutation of *DYNC2H1* may be associated with severe short limbs and heart malformations in SRTD3. In our study, the phenotype of the four fetuses were also inconsistent. For example, the phenotype of fetus 1 included multiple anomalies of the fetus, including short long bones and narrow thorax. The phenotype of fetus 2 contained short long bones, seroperitoneum and polyhydramnios. The phenotype of fetus 3 involved severe short limbs, abnormal posture of hand and foot, cardiac malformation and single umbilical artery. The phenotype of fetus 4 concerned short femur length and slightly bending. In addition to fetus 3, the SRTD3 phenotype of the other three cases was relatively lighter. In conclusion, we speculate that different mutation sites of *DYNC2H1* may produce different SRTD3 phenotypes. Therefore, it is important to further explore the effects of different sites on SRTD3 phenotypes.

Rapid advances in high-throughput sequencing and bioinformatics technology have allowed it possible to reveal how disease mutations disrupt protein-protein interaction (PPI) networks in human cells (Cheng et al., 2021). It is well known that protein-protein interaction network plays a crucial role in all biological processes (Sneha et al., 2018). Protein-protein interaction networks have been used to study the pathogenesis and potential pathogenic molecules of many diseases (Röhl et al., 2022; Vagiona et al., 2022). In our study, the protein-protein interaction network was consisted of 11 nodes and 51 edges. Then, these 11 genes were utilized to perform the GO enrichment analysis. GO terms enrichment analysis showed that ciliary tip, intraciliary transport, intraciliary retrograde transport, cytoplasmic dynein complex, intraciliary transport particle, ciliary plasm, centrosome, ciliary basal body, appendage development and motile cilium were significantly enriched GO terms. All these GO processes are biological processes related to cilia function. SRTD3 has been widely recognized as a typical ciliary disease, and its characteristics overlap with the molecular mechanisms of ciliary dysfunction (Taylor et al., 2015). Therefore, we speculated that these 11 genes may participate in the SRTD3 progression by regulating these GO processes.

Several studies have found that genes splicing mutations can affect phenotype and disease risk (Manning and Cooper, 2017; Park et al., 2018). These studies have broad implications as they can shed light on disease pathogenesis and inform treatment options for the disease (Matesanz et al., 2015; Chen et al., 2021). In regard to the c.8833-1G > A variant, the vitro minigene splice assay confirmed that the variant caused exon 56 skipping, suggesting that this variant could increase the use of PS3 evidence. On the basis of the ACMG/AMP guidelines, c.8833-1G > A variant was classified as likely pathogenic with the criteria of PS3+PVS1_S + PM2_Supporting.

## Conclusion

The present study built the genetic diagnosis on four families affected with SRTD3 by WES analysis, and identified eight *DYNC2H1* variants, which expanded its mutation spectrum. The results of our study offer a strong theoretical foundation for genetic counseling in affected families and provide a basis for elucidating importance of *DYNC2H1* function in the pathogenesis of SRTD3. The next functional studies will contribute to uncover the potential pathogenesis of SRTD3 induced by *DYNC2H1* gene variants and offer potential therapeutic ways in the future.

## Data Availability

The data presented in this study are deposited in Sequence ReadArchive (SRA) repository, accession number PRJNA935106.

## Supplementary material

Supplementary Figure S1 The workflow of the variant calling, annotating and filtering strategy in this study.

Supplementary Figure S2 Genotypic data. Whole-exome data using BAM files in the Integrated Genomics Viewer (IGV) depicting the genotypic information of *DYNC2H1* BAM variant in family 1 samples. **(A)** c.3842A>C. **(B)** c.8833-1G>A.

Supplementary Figure S3 Genotypic data. Whole-exome data using BAM files in the Integrated Genomics Viewer (IGV) depicting the genotypic information of *DYNC2H1* BAM variant in case 2 samples. **(A)** c.8617A>G. **(B)** c.7053_7054del.

Supplementary Figure S4 Genotypic data. Whole-exome data using BAM files in the Integrated Genomics Viewer (IGV) depicting the genotypic information of *DYNC2H1* BAM variant in family 3 samples. **(A)** c.5984C>T. **(B)** c.10219C>T.

Supplementary Figure S5 Genotypic data. Whole-exome data using BAM files in the Integrated Genomics Viewer (IGV) depicting the genotypic information of *DYNC2H1* BAM variant in family 4 samples. **(A)** c.5256del. **(B)** c.9737C>T.

Supplementary Table S1 Detailed variants list of case 1 observed in WES.

Supplementary Table S2 Detailed variants list of case 2 observed in WES.

Supplementary Table S3 Detailed variants list of case 3 observed in WES.

Supplementary Table S4 Detailed variants list of case 4 observed in WES.
