# Supplementary figures and images for: Genetic analysis and prenatal diagnosis of short-rib thoracic dysplasia 3 with or without polydactyly caused by compound heterozygous variants of DYNC2H1 gene in four Chinese families
Source: Front Genet. 2023 Mar 17;14:1075187. doi: 10.3389/fgene.2023.1075187 (PMC10064095; doi:10.3389/fgene.2023.1075187)

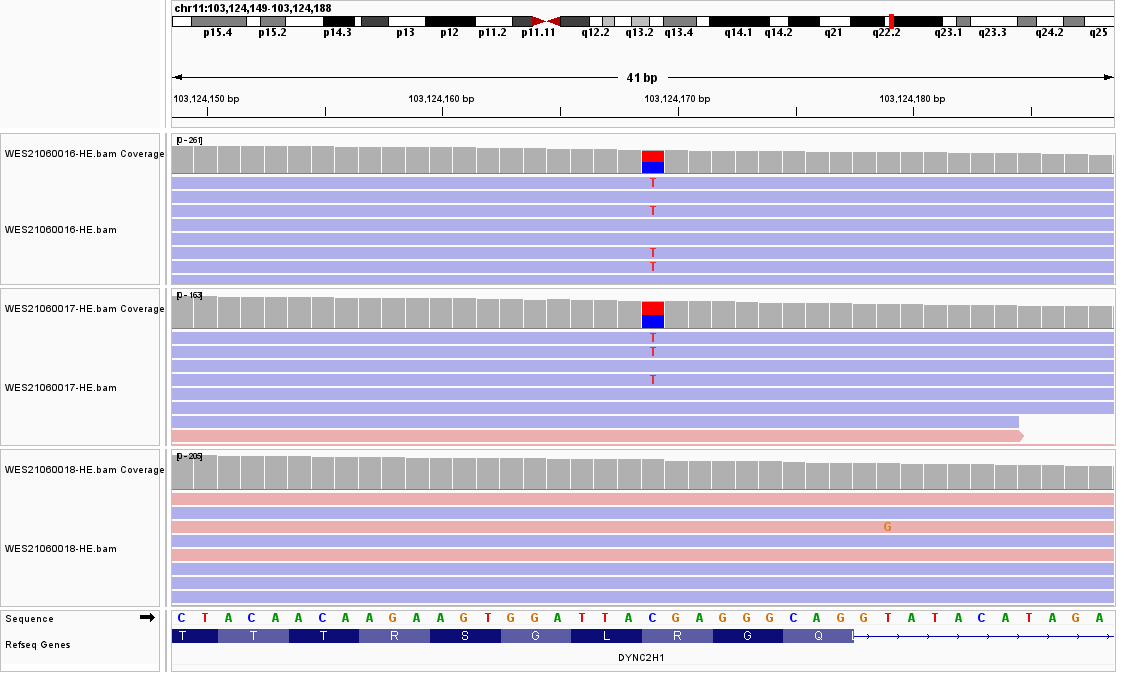

Supplement: Supplementary file 5 [file Image6.TIF]

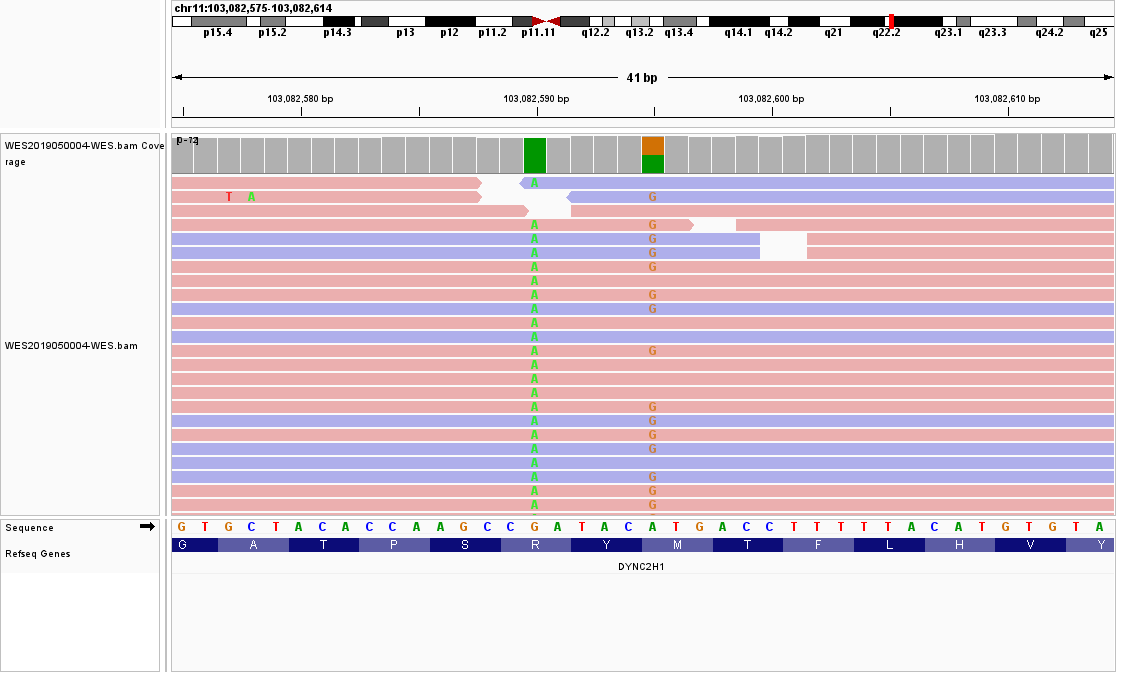

Supplement: Supplementary file 6 [file Image3.TIF]

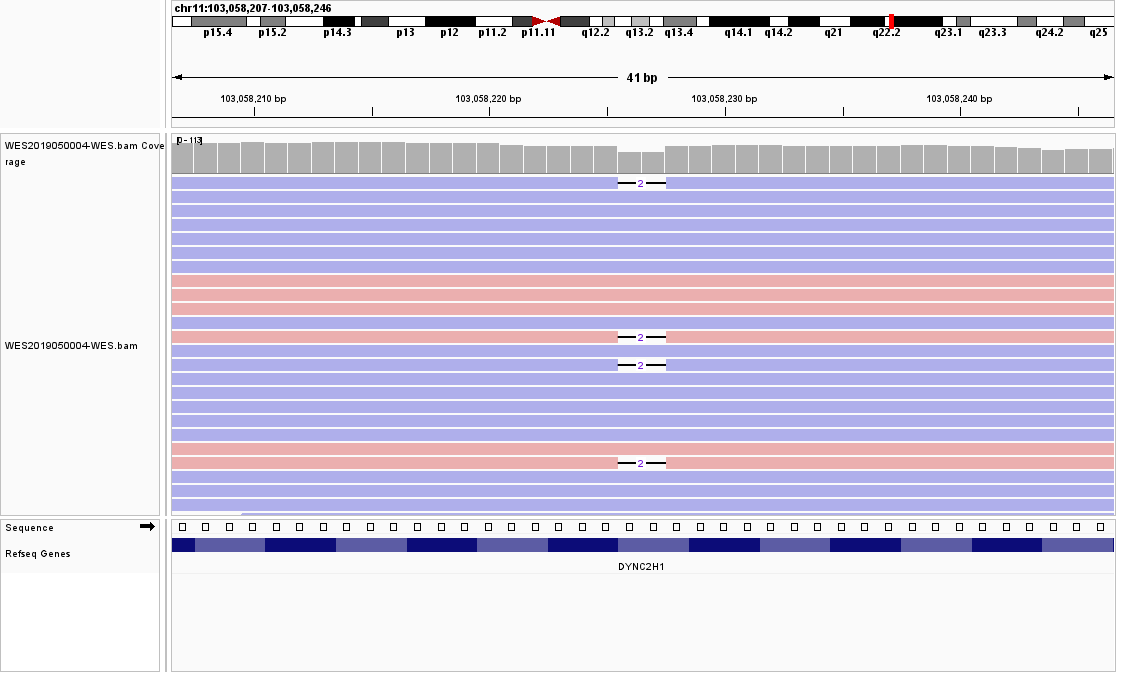

Supplement: Supplementary file 7 [file Image4.TIF]

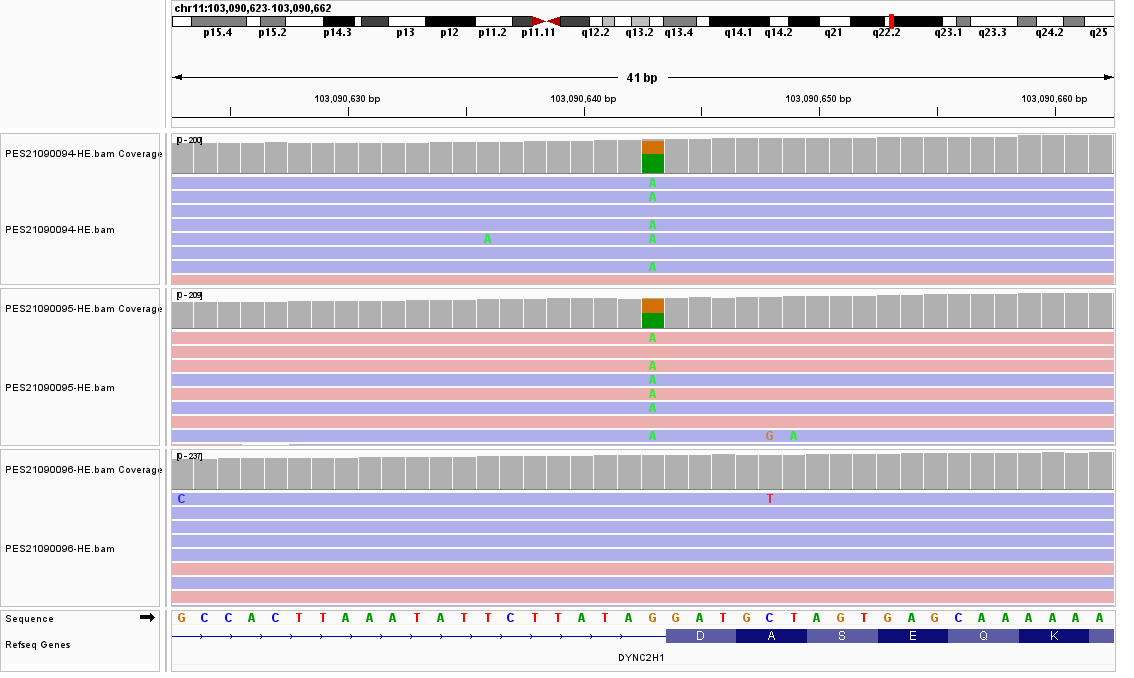

Supplement: Supplementary file 8 [file Image2.TIF]

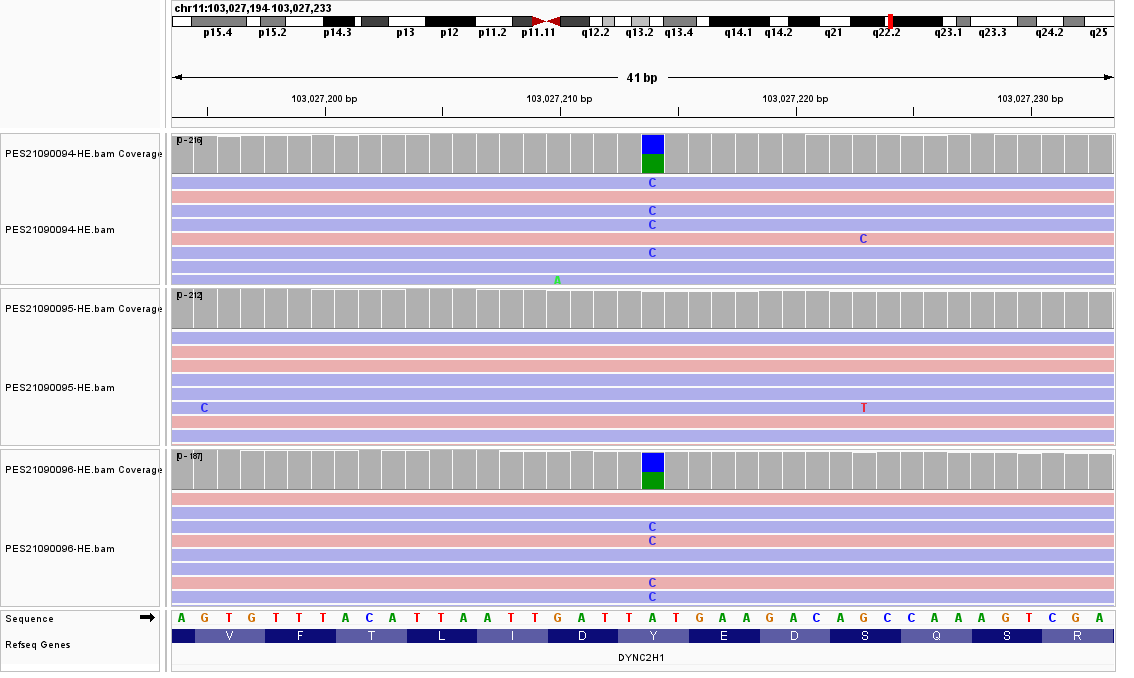

Supplement: Supplementary file 9 [file Image1.TIF]

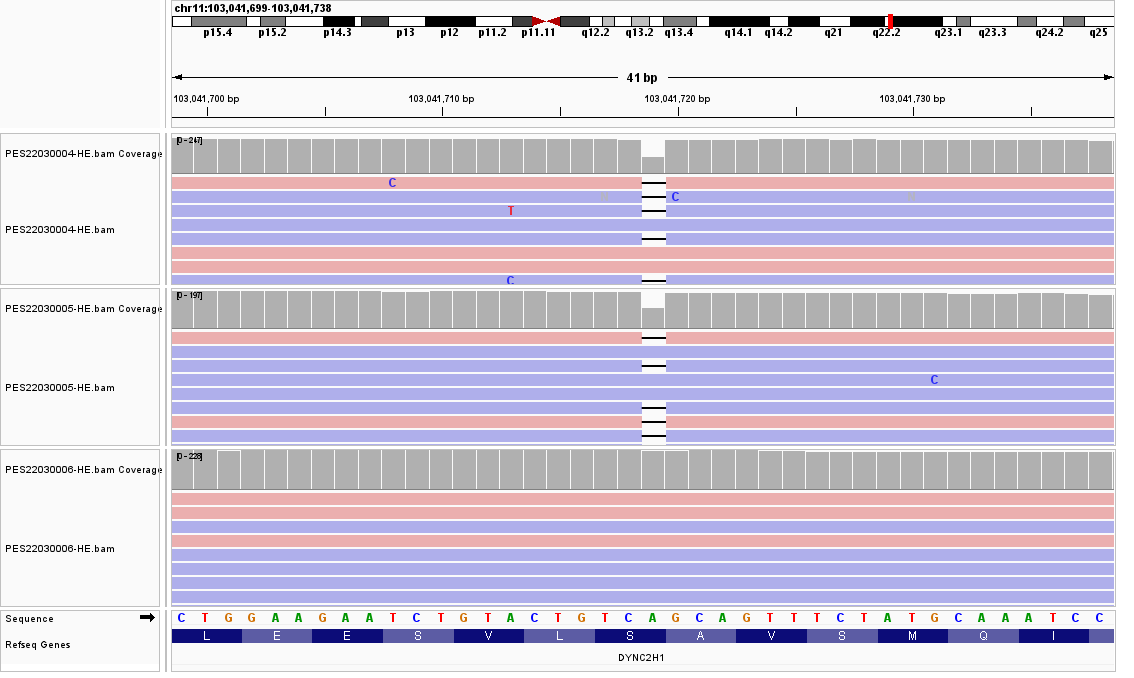

Supplement: Supplementary file 10 [file Image7.TIF]

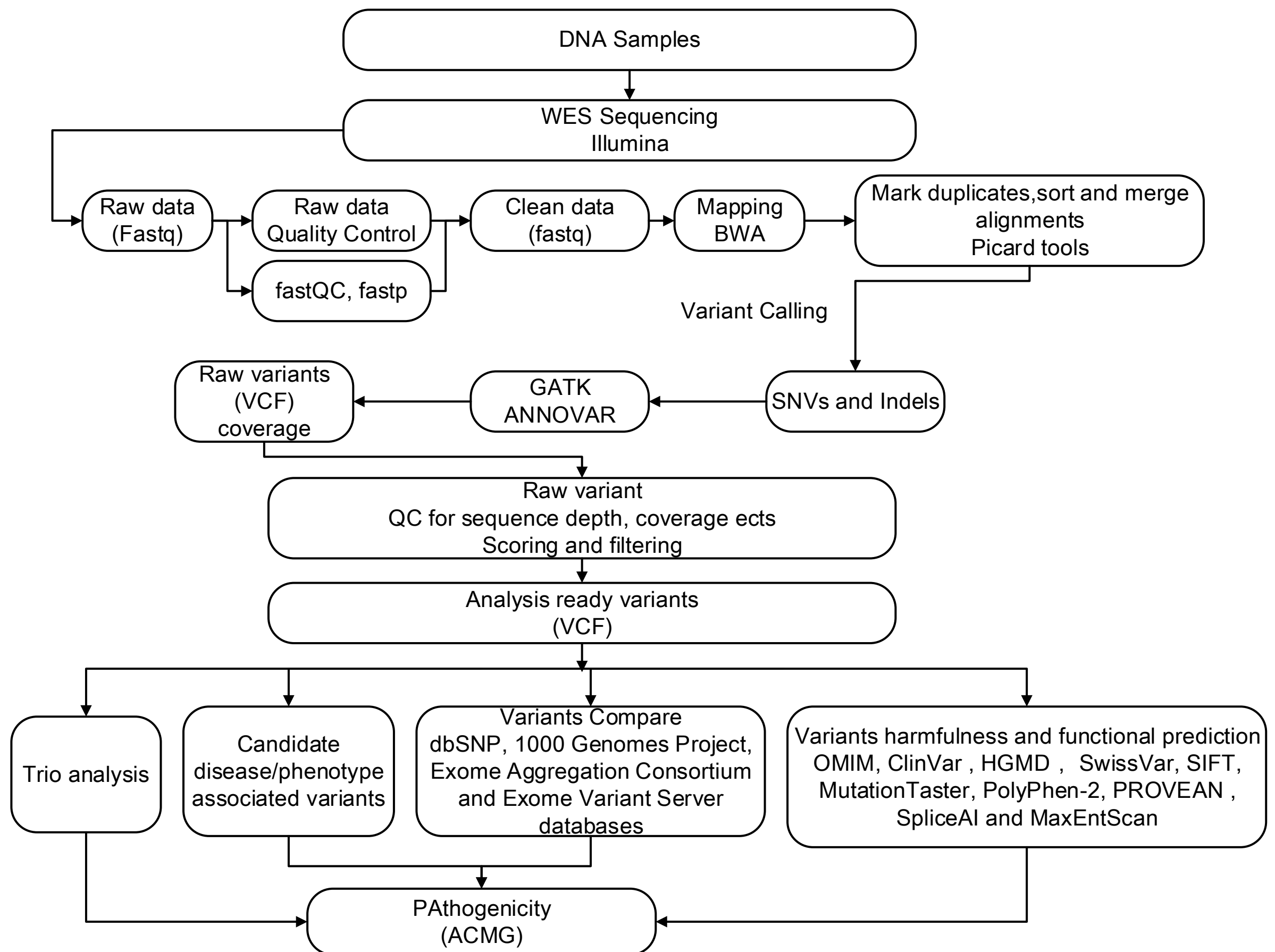

Supplement: Supplementary file 11 [file DataSheet1.PDF]

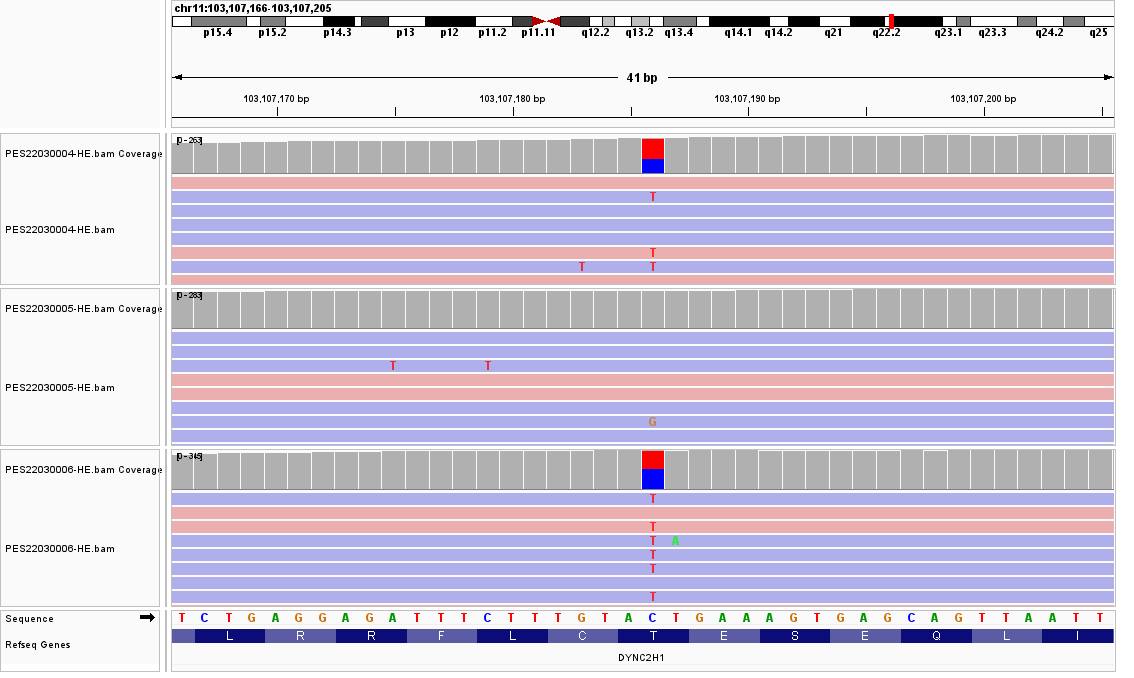

Supplement: Supplementary file 12 [file Image8.TIF]

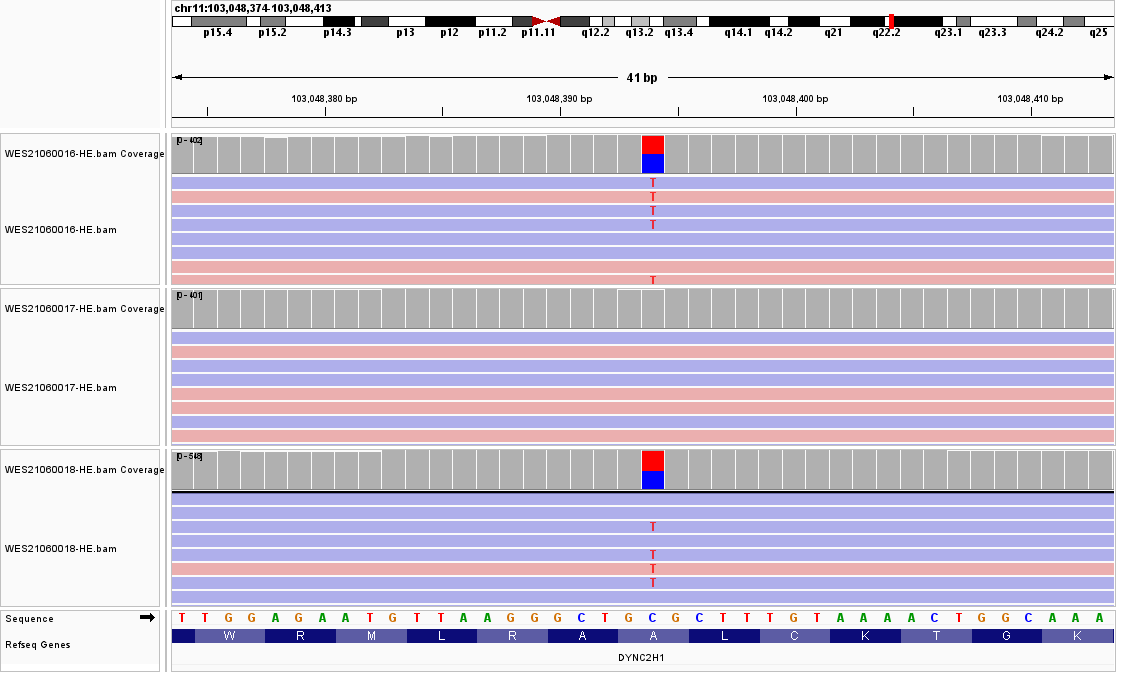

Supplement: Supplementary file 13 [file Image5.TIF]
